# Nematode-Associated Bacteria: Production of Antimicrobial Agent as a Presumptive Nominee for Curing Endodontic Infections Caused by *Enterococcus faecalis*

**DOI:** 10.3389/fmicb.2019.02672

**Published:** 2019-11-22

**Authors:** Hicran Donmez Ozkan, Harun Cimen, Derya Ulug, Sebastian Wenski, Senem Yigit Ozer, Murat Telli, Neriman Aydin, Helge B. Bode, Selcuk Hazir

**Affiliations:** ^1^Department of Endodontics, Faculty of Dentistry, Adnan Menderes University, Aydin, Turkey; ^2^Department of Biology, Faculty of Arts and Sciences, Adnan Menderes University, Aydin, Turkey; ^3^Molekulare Biotechnologie, Fachbereich Biowissenschaften, Buchmann Institute for Molecular Life Sciences (BMLS), Goethe Universität Frankfurt Biozentrum, Frankfurt am Main, Germany; ^4^Department of Microbiology, Faculty of Medicine, Adnan Menderes University, Aydin, Turkey

**Keywords:** endodontic infections, Enterococcus faecalis, fabclavine, *Photorhabdus*, *Xenorhabdus*

## Abstract

*Xenorhabdus* and/or *Photorhabdus* bacteria produce antibacterial metabolites to protect insect cadavers against food competitors allowing them to survive in nature with their nematode host. The effects of culture supernatant produced by *Xenorhabdus* and *Photorhabdus* spp. were investigated against the multidrug-resistant dental root canal pathogen *Enterococcus faecalis.* The efficacy of seven different cell-free supernatants of *Xenorhabdus* and *Photorhabdus* species against *E. faecalis* was assessed with overlay bioassay and serial dilution techniques. Additionally, time-dependent inactivation of supernatant was evaluated. Among the seven different bacterial species, *X. cabanillasii* produced the strongest antibacterial effects. Loss of bioactivity in a phosphopantetheinyl transferase-deficient mutant of *X. cabanillasii* indicated that this activity is likely based on non-ribosomal peptide synthetases (NRPSs) or polyketide synthases (PKSs). Subsequent *in silico* analysis revealed multiple possible biosynthetic gene clusters (BGCs) in the genome of *X. cabanillasii* including a BGC homologous to that of zeamine/fabclavine biosynthesis. Fabclavines are NRPS-derived hexapeptides, which are connected by PKS-derived malonate units to an unusual polyamine, also PKS-derived. Due to the known broad-spectrum bioactivity of the fabclavines, we generated a promoter exchange mutant in front of the fabclavine-like BGC. This leads to over-expression by induction or a knock-out by non-induction which resulted in a bioactive and non-bioactive mutant. Furthermore, MS and MS^2^ experiments confirmed that *X. cabanillasii* produces the same derivatives as *X. budapestensis*. The medicament potential of 10-fold concentrated supernatant of induced fcl promoter exchanged *X. cabanillasii* was also assessed in dental root canals. Calcium hydroxide paste, or chlorhexidine gel, or fabclavine-rich supernatant was applied to root canals. Fabclavine-rich supernatant exhibited the highest inactivation efficacy of ≥3 log_10_ steps CFU reduction, followed by calcium hydroxide paste (≤2 log_10_ step). The mean percentage of *E. faecalis*-free dental root canals after treatment was 63.6, 45.5, and 18.2% for fabclavine, calcium hydroxide, and chlorhexidine, respectively. Fabclavine in liquid form or preferably as a paste or gel formulation is a promising alternative intracanal medicament.

## Introduction

*Enterococcus faecalis* is a species of the Enterococci that is associated with humans as part of the microbiota of the gastrointestinal system. However, the bacterium sometimes is an opportunistic human pathogen (Karchmer, 2000). *E. faecalis* is the most common etiological agent of human enterococcal infections (Kayaoglu and Orstavik, 2004). Moreover, this bacterium can exist as a nosocomial infection and result in mortalities surpassing 50% in some immunocompromised and cancer patients (Schmidt-Hieber et al., 2007; Arias and Murray, 2012).

In dentistry, *E. faecalis* is linked to persistent periradicular lesions with major endodontic infections and persistent infections of the root canal (Sanchez-Sanhueza et al., 2015). Eradication of *E. faecalis* is challenging because it creates a biofilm, utilizes diverse compounds as energy sources, and survives extreme environmental conditions (Gilmore et al., 2002; Tendolkar et al., 2003). These characteristics contribute to bacterial tenacity and virulence in tooth infections (Stojicic et al., 2010).

The number of bacterial cells can be reduced by shaping of the root canal with mechanical instrumentation and irrigation with antimicrobial agents. However, these techniques are inept in adequately eliminating *E. faecalis* due to the complex anatomy of the root canal system (Stuart et al., 2006; Vianna and Gomes, 2009; Asnaashari et al., 2017). Accordingly, intracanal treatment is recommended for lowering the number of bacteria before filling the root canal (Bystrom et al., 1985). Calcium hydroxide (Ca(OH)_2_) pastes and chlorhexidine (CHX) gels are commonly used as intracanal medicaments (Siqueira and de Uzeda, 1997; Tervit et al., 2009).

Even though CHX and Ca(OH)_2_ are regular intracanal medicaments in endodontic therapy, previous studies have revealed that *E. faecalis* can still persist (Orstavik and Haapasalo, 1990; Heling et al., 1992; Evans et al., 2002; Delgado et al., 2010). Moreover, an effective antibiotic to decolonize patients with antibiotic-resistant *E. faecalis* is unknown or unavailable. The health care concern posed by *E. faecalis* stresses the pressing urgency for new approaches for decolonization and therapeutic treatment. The discovery of novel antibiotics or antibacterial agents can serve as alternatives for *E. faecalis* suppression. The most important antibacterial sources in nature are viruses (Suttle, 1994; Fuhrman, 1999), fungi (Brian and Hemming, 1947; Zhang et al., 2012), bacteria (Kirkup, 2006; Gillor and Ghazaryan, 2007; Newman and Cragg, 2016), and plants (Cowan, 1999). Various fungi and bacteria produce antimicrobial compounds as secondary metabolites to compete with other organisms. Among bacteria, research over the past three decades has shown that the genera *Photorhabdus* and *Xenorhabdus* produce antimicrobial compounds that may have potential use against an array of bacterial pathogens (Boemare and Akhurst, 2006; Shi and Bode, 2018).

*Xenorhabdus* and *Photorhabdus* species are insect pathogenic bacteria that are symbiotically associated with nematodes in the families Steinernematidae and Heterorhabditidae, respectively (Hazir et al., 2003). These entomopathogenic nematodes (EPNs) with their bacteria are obligate, lethal parasites of soil insects. The symbiotic organisms have many positive attributes such as safety to humans and nontarget organisms and ease of mass production (Fodor et al., 2017).

The nematode-killed insect is protected from secondary invasion by contaminating organisms that allow the nematodes to develop in the cadaver. The protection is provided by *Photorhabdus* and *Xenorhabdus* by the production of a variety of small antibiotic molecules. For example, *Xenorhabdus* spp. synthesize a variety of secondary metabolites including antimicrobials made of linear and cyclic peptides (Bode, 2009; Shi and Bode, 2018). To date, the compounds examined from *X. bovienii, X. nematophila,* and *X. cabanillasii*, are indole, xenorhabdin, xenocoumacin, PAX peptides, and cabanillasin with antibacterial, antifungal, or both activities (Gu et al., 2009; Hazir et al., 2016). It was stated that *Photorhabdus* species also generate antimicrobial compounds including isopropylstilbenes and the β-lactam carbapenem (Webster et al., 2002). Some of these compounds especially from *P. temperata* and *P. luminescens* subsp. *luminescens* are known to have antibiotic properties. The trans-stilbenes and anthraquinone pigments were detected as antibacterial (Boemare and Akhurst, 2006). These findings of antimicrobial compounds have attracted considerable interest for pharmaceutical and agronomic purposes (Webster et al., 2002; Hazir et al., 2016). It is known that different species/strains of *Xenorhabdus* and *Photorhabdus* produce various antimicrobial compounds. Hence, we hypothesized that some species of *Xenorhabdus* and/or *Photorhabdus* spp. produce active compound(s) in their secondary metabolites that will inhibit the growth of antibiotic-resistant *E. faecalis*. Accordingly, we tested this hypothesis with seven different supernatants of *Xenorhabdus* and *Photorhabdus* species against antibiotic-resistant *E. faecalis* with *in vitro* antibacterial tests. Subsequently, the medicament potential of the antibacterial compound obtained from the most effective species was compared with CHX and Ca(OH)_2_ against *E. faecalis* in root canals.

## Materials and Methods

### Source of Bacteria and Supernatant Preparation

Antibiotic activity of seven bacterial isolates of *Xenorhabdus* and *Photorhabdus* was tested against *E. faecalis*. The nematode species and strains from which each tested bacterial species or subspecies was isolated is presented in Supplementary Table S1. Henceforth, we will refer to all isolates as bacterial species even though we recognize that *P. luminescens* includes two subspecies (*luminescens* and *laumondii*).

Bacterial isolates were recovered from nematode-infected *Galleria mellonella* (Lepidoptera: Pyralidae) larva (Kaya and Stock, 1997). *Xenorhabdus* and *Photorhabdus* bacteria have phase-changing capabilities when cultured *in vitro*. Phase-I is associated with nematodes and produces toxins, enzymes, antibiotics, etc. that provide better support for nematode growth in insect cadavers, whereas phase-II occurs spontaneously under unfavorable conditions or long incubation periods (Leclerc and Boemare, 1991; Boemare, 2002). Thus, we used only phase-I of each of the bacterial isolates as observed by their distinctive colony and cell morphology on NBTA (nutrient agar 31 g/L, bromothymol blue 25 mg/L, and 2,3,5-triphenyl tetrazolium chloride 40 mg/L) plates and by a catalase test. After isolating the phase-I bacterium, each isolate was stored at −80°C until further use (Boemare and Akhurst, 2006).

To conduct the experiments, each bacterial isolate was streaked onto a NBTA plate and after 24 h, a loopful of bacterial cells was transferred to 100 ml of Tryptic Soy Broth (TSB) (Difco, Detroit, MI) in an Erlenmeyer flask. Because the optimum time for antibiotic production is 120–144 h (Furgani et al., 2008), cultures were incubated at 28°C and 150 rpm for 144 h (Hazir et al., 2016). Later on, supernatants were obtained from the centrifuged bacterial culture at 4°C and 20,000 *g* for 15 min. To eliminate all bacterial cells, the supernatants were filtered through a 0.22-μm Millipore filter (Thermo scientific, NY). Each cell-free supernatant was stored at 4°C in sterile falcon tubes (Corning, NY) and used within 2 weeks.

### Pathogen Cultures

Multidrug-resistant *Enterococcus faecalis* V583 (ATCC 700802) was used for the experiments. The strain was grown overnight in Trypticase Soy broth (TSB) (Merck) (Awori et al., 2016) and the bacterial stock suspension was kept at −80°C (Boemare and Akhurst, 2006).

### Antibacterial Activity of Different *Photorhabdus* and *Xenorhabdus* Spp. Against *Enterococcus faecalis*

#### Overlay Bioassay

Cell-to-cell competition of test bacteria with the antibiotic producer colony on a solid media (slightly modified method of Furgani et al., 2008) was used for the antibacterial test. The overlay bioassay permitted us to assess the efficacy of different species of antibiotic producing *Xenorhabdus* and *Photorhabdus* spp. against *E. faecalis* by measuring the inactivation zones. *Xenorhabdus* and *Photorhabdus* cultures were prepared by inoculating a loopful of bacteria from NBTA plate into 50 ml of TSB and incubating them at 150 rpm and 28°C in an incubator overnight. A 5-μl bacterial sample from the overnight culture was transferred onto the center of Mueller Hinton Agar (MHA) (Merck) plates. The bacteria were incubated for 5 days at 28°C (Furgani et al., 2008). For preparation of overnight *E. faecalis* culture*, E. faecalis* was inoculated in 50 ml of TSB medium and incubated at 37°C and 150 rpm. A volume of 100 μl of the pathogen culture (44 × 10^8^ CFU/ml) was added to 3.5 ml of soft agar (0.6% w/v) at 45–50°C, which was then poured into the test plates where the *Xenorhabdus* or *Photorhabdus* colony had been growing. To prevent bacterial expansion on agar media, the propagated *Xenorhabdus* or *Photorhabdus* colony was left under UV light for 5 min before layering the mixture of *E. faecalis* and soft agar over the plate. After the solidification of soft agar, the petri dishes were incubated for 48 h at 37°C. The zone diameter around the colony of antibiotic-producing cells was measured in two directions perpendicular to each other and the average was taken (Mattigatti et al., 2012). Each bacterial species had 10 replicates and the experiment was conducted three times.

### Antibacterial Activity of Cell-Free *Xenorhabdus* or *Photorhabdus* Supernatants

The cell-free supernatants of all species of *Xenorhabdus* and *Photorhabdus* listed in Supplementary Table S1 were tested against *E. faecalis.* Different proportions (1, 5, 10, 20, 30, 40, 50, and 100%) of supernatants containing bacterial metabolites were tested for inactivation of growth of *E. faecalis*. For each proportion (1, 5, 10, 20, 30, 40, 50, and 100%), filtrated supernatants were incorporated on a *v/v* basis into test tubes with 2 ml of sterile Mueller Hinton Broth (MHB) (Merck) (Gualtieri et al., 2012). According to supernatant proportions, the same amount of MHB was discarded before adding bacterial supernatant (for example, for 5%, 0.1 ml supernatant was incorporated into 1.9 ml of MHB). A volume of 10 μl of a culture of *E. faecalis* (44 × 10^8^ CFU/ml) incubated overnight was pipetted into the test tubes containing MHB and cell-free supernatants. There were positive and negative control groups. Positive control included MHB and *E. faecalis*, whereas the negative control was only cell-free supernatant. The tubes were incubated in the shaker incubator at 150 rpm and 37°C for 48 h. Following the incubation period, bacterial growth was evaluated visually to determine maximum inhibiting dilutions (MIDs; according to Furgani et al., 2008, we used the term “dilution” not concentration). The MID is the maximum supernatant dilution that yields no visible growth. In each series of tubes, the last tube with clear supernatant was considered to be without any growth and was assumed to give the MID value. Turbidity in the tubes indicated growth of *E. faecalis*. Visual evaluations were made independently by three examiners and a consensus opinion was agreed upon (Furgani et al., 2008; Aarati et al., 2011). An aliquot of 100 μl was taken from each tube where no bacterial growth had been observed visually and was transferred to the blood-agar medium (5% sheep blood) to determine maximum bactericidal dilution (MBD). After streaking the subsamples on the blood-agar medium, the petri dishes were incubated at 37°C for 48 h to verify total inactivation. The smaller the MID or MBD values, the stronger the antibiotic production obtained (Furgani et al., 2008). National Committee for Clinical Laboratory Standart Institute (CLSI) recommended procedures were used for MID and MBD determination.

Three replicates were used for each supernatant proportion and the experiment was conducted three times.

### Time-Dependent Inactivation of Cell-Free Supernatant

Depending on the results of the experiment on overlay bioassay and the MID and MBD values, bacterial supernatant that produced the maximum antibacterial activity (*X. cabanillasii* supernatant) was used to determine the time to inactivation of *E. faecalis*. This was done by using 25 ml of sterile TSB mixed with 25 ml of cell-free supernatant of *X. cabanillasii* in a 100-ml flask. A 0.5-ml aliquot of *E. faecalis* from an overnight culture (44 × 10^8^ CFU/ml) was transferred to the 50% supernatant, and afterward the flask was incubated at 37°C at 150 rpm. On a 2-h basis from 0 to 16 h, a 10-μl subsample was pipetted from the flask and spread on a blood agar. Plates were incubated for 48 h at 37°C. Three replicates were used and the experiment was conducted three times.

### Identification of Bioactive Antibacterial Compound

To determine the bioactive compound, promoter exchanged mutants of *X. cabanillasii* were generated and matrix assisted laser desorption/ionization-mass spectrometry (MALDI-MS) and MALDI-MS^2^ experiments were performed.

### Generation of Deletion and Promoter Exchange Mutants in *Xenorhabdus cabanillasii*

Due to the phosphopantetheinyl transferase (PPTase)-dependence of NRPS- and PKS-derived secondary metabolites, we deleted the responsible gene in *X. cabanillasii*. This should lead to a loss of production and bioactivity in the mutant if the compound is NRPS- or PKS-derived.

Deletion of the phosphopantetheinyl transferase (Xcab_04003) in *X. cabanillasii* was performed by double homologous recombination. About 1,000 bps were amplified by PCR with the primers SW305_Xcab_LF_fw (5′-CGATCCTCTAGAGTCGACCTGCAGTGTATAGGTCATAGCGCATTTTCC-3′) and SW306_Xcab_LF_rv (5′-TTTCATCTCTTATTTTGTTGTTCTTGGGTATTGTTCG-3′) for the upstream and SW307_Xcab_RF_fw (5′-TACCCAAGAACAACAAAATAAGAGATGAAAACCCCGG-3′) and SW308_Xcab_RF_rv (5′-GAGAGCTCAGATCTACGCGTTTCATATGGGTTTTAGCCCAATCTTATGCC-3′) for the downstream regions. Both were integrated by Hot Fusion assembly into the *Pst*I/*Nde*I digested deletion vector pDS132 and transformed into *E. coli* ST18 (Fu et al., 2014). The plasmid pDS132 containing the *sacB* gene and a kanamycin resistance cassette. *X. cabanillasii* was conjugated with *E. coli* ST18 and insertion mutants were selected on kanamycin-supplemented LB agar plates. The second homologous recombination was then enforced by cultivation on sucrose-supplemented LB agar plates, which is toxic due to the SacB conversion.

Promoter exchange in front of the fcl-homologous gene cluster was performed upstream of the *fclC*-like gene (Xcab_02060) in *X. cabanillasii*. The first 1,000 bps of Xcab_02060 were amplified by the primers SW128_Xcab_fw (5′-TTTGGGCTAACAGGAGGCTAGCATATGACCAAGACGTATTTTTTGCATG-3′) and SW129_Xcab_rv (5′-TCTGCAGAGCTCGAGCATGCACATTTTACCTGCCCTTCCAGACG-3′) and cloned into the PCR-amplified vector pCEP_kan by Hot Fusion assembly (Fu et al., 2014; Bode et al., 2015). After transformation, positive *E. coli* S17 clones were confirmed by restriction, conjugated with *X. cabanillasii,* and insertion mutants selected on kanamycin-supplemented LB agar plates (Supplementary Table S2). Successfully generated deletion and promoter exchange mutants were verified by colony PCR and analyzed by MALDI-MS.

### Identification of Antibacterial Compound by MALDI-MS and MALDI-MS^2^

Cultures for MALDI-MS measurements were prepared with 10 ml of lysogeny broth media supplemented with kanamycin (50 μg/ml) if appropriate, inoculated with 400 μl of a preculture, and incubated at 30°C for 72 h with shaking. Induced promoter exchange mutants were additionally supplemented with 0.2% L-arabinose (Bode et al., 2015). Liquid cultures were spotted on a steel target with a volume of 0.3 μl mixed with 0.25 μl of 1:10 diluted ProteoMass Normal Mass Calibration Mix (Sequazyme™ Peptide Mass Standards Kit) for internal calibration and 0.9 μl of alpha-Cyano-4-hydroxycinnamic acid (CHCA) matrix (3 mg/ml in 75% acetonitrile, 0.1% trifluoroacetic acid). After air-drying, the sample spot was washed with 5% formic acid and mixed again with 0.6 μl of CHCA. Cell MALDI measurements were performed with a MALDI LTQ Orbitrap XL (Thermo Fisher Scientific, Inc., Waltham, MA) instrument with a nitrogen laser at 337 nm in FTMS scan mode with 100 shots per measurement in a mass range of 350–1,500 m/z with high resolution. MALDI-MS^2^ experiments were performed in CID-mode using ITMS scan mode with the following parameters: Normalized collision energy, 28; Act. Q, 0.250; and Act. Time (ms), 30.0. Data were analyzed using Qual Browser version 2.0.7 (Thermo Fisher Scientific, Inc., Waltham, MA).

### Testing the Antibacterial Activity of *Xenorhabdus cabanillasii* Mutant Strains

Antibacterial activity of 5-day-old cell-free supernatants of wild-type, pptase deletion mutant, induced (with arabinose) and non-induced (without arabinose) *fclC* promoter exchange mutant of *X. cabanillasii* was tested with agar-well diffusion bioassay as described before. Each petri dish included four wells and each well was filled with 70 μl of one of the cell-free *X. cabanillasii* supernatants. After the incubation period for 48 h at 37°C, the inactivation zones (mm) on plates were measured (Mattigatti et al., 2012). Five replicates were used and the experiment was conducted three times.

### Medicament Potential of the Antibacterial Compound in Dental Root Canals

One-day-old cell-free supernatant of induced *fcl*C mutant strain of *X. cabanillasii* was concentrated 10-fold using an evaporator and tested in dental root canals. Recently extracted human mandibular premolars were collected from patients who signed a patients’ consent protocol (2016/1052) approved by Adnan Menderes University, Local Ethical Committee. Soft tissue remnants and calculus were removed from the external root surfaces by using a periodontal scaler. Periapical radiographs were taken from both buccolingual and mesiodistal directions to determine the teeth that have a straight single root canal. Next, a dental operating microscope (Leica M320) was used to select the teeth with no resorption, defect, or cracks. According to these criteria, 44 mandibular premolar teeth with curvature less than 5° and 15–18 mm long were selected (Schneider, 1971; Pladisai et al., 2016). The roots were sectioned using a diamond disk, perpendicular to the long axis into samples 13 mm long from the cementoenamel junction to the apical root end (Pladisai et al., 2016). A single endodontist removed the pulp tissue and checked the canal patency with a #10 stainless steel K-File (Mani Inc. Tochigi, Japan) until it was visible at the apical foramen. Working length was set at 1 mm short of this length. Then root canals were instrumented with Protaper Next rotary system (Dentsply, Ballaigues, Switzerland) up to X3 (0.30 mm tip with 7% taper) by the same endodontist in a crown-down manner, at a rotational speed of 300 rpm and 200 g/cm torque. Root canals were washed with 2 ml of 5% NaOCl using a 27-gauge notched-tip irrigation needle (Ultradent, UT, USA) between each instrument. At the end of the instrumentation, a final flush was applied using a sequence of 5 ml of 17% EDTA and 5 ml of 5% NaOCl to remove the smear layer, followed by 5 ml of 10% sodium thiosulfate and 5 ml of sterile distilled water (Sasanakul et al., 2019). Finally, specimens were dried with paper points. All teeth were sterilized in an autoclave (121°C for 15 min) before using in the experiments (Alves et al., 2013; Zan et al., 2013).

A 10-μl aliquot of *E. faecalis* from an overnight culture adjusted to 5 × 10^5^ CFU/ml (Ardizzoni et al., 2009) was placed in the root canals of 44 teeth using a sterile micropipette. The teeth were placed individually in 2-ml capacity sterile centrifuge tubes containing 1 ml of BHI broth and incubated for 21 days at 37°C. This is to allow bacteria to penetrate the dentinal tubules and biofilm formation (Pladisai et al., 2016). The media were replaced with sterile BHI every other day (Sasanakul et al., 2019).

After the incubation period, the teeth were embedded in the silicone impression material to create a closed-end channel (Tay et al., 2010). Then, to imitate the final irrigation process before medicament applications in clinical conditions, the specimens were irrigated with 3 ml of 17% EDTA, 3 ml of 5% NaOCl, 3 ml of 10% sodium thiosulfate, and 3 ml of sterile distilled water, respectively. The root canals were subsequently dried with paper points and they were randomly divided into four groups (*n* = 11 specimens per group).

The root canals of the first and second groups were filled with Ca(OH)_2_ paste (Ultracal XS, Ultradent Products Inc., USA) and 2% CHX gluconate gel (Gluco-Chex, Cerkamed, Poland) by using a Lentulo spiral (Dentsply-Maillefer), respectively. The root canals in the third group were filled with 10-fold concentrated supernatant of *fclC*-induced *X. cabanillasii* with the help of a sterile syringe. The remaining 11 teeth were filled with sterile BHI medium and this served as the control group. Coronal access of the teeth was closed with parafilm. After the treatments, silicon impression material around the specimens was removed and it was placed in 2-ml sterile centrifuge tubes containing BHI medium individually. The tubes were incubated aerobically at 37°C for 7 days. The media were replaced with sterile BHI every other day.

At the end of experimental period, a ProTaper X3 rotary instrument was used for 20 s to remove CHX gel and Ca(OH)2 paste. For the neutralization of Ca(OH)2, 3 ml of 5% citric acid, and for CHX gel, the mixture of 0.3% L-α-Lecithin and 3% Tween 80 were used. Then both medicament groups were irrigated with 3 ml of physiological saline (Pektas et al., 2013). The specimens in supernatant applied and control group were treated with 6 ml of sterile physiological saline.

The remaining bacteria in the root canals and inner dentins were collected by shaving the root canal walls with a No. 4 Peeso reamer (Dentsply-Maillefer). The collected dentin chips were transferred to 0.5 ml of BHI medium in Eppendorf tube and vortexed vigorously. Then, 50-μl sample was streaked on blood-agar plates. The plates were incubated at 37°C for 48 h and the colonies on blood agar were counted and inferred as colony forming units (CFUs).

### Statistical Analysis

SPSS 25.0 (IBM Corp., Chicago, IL, USA) package program with a level of significance set at 0.05 was used. Differences in antibacterial effects of the supernatants were compared with one-way ANOVA and the means separated using Tukey’s test. The data of time-dependent CFU reduction and medicament potential of the antibacterial compound in dental root canals were shown as medians, including 25 and 75% quartiles. In these results, horizontal dotted, solid, and dashed lines represent reductions of 2, 3, and 5 log_10_ steps CFU, respectively. Medians on or below these lines exhibit bacterial killing efficacy of 99% (2 log_10_), 99.9% (3 log _10_), and 99.999 (5 log _10_) (Boyce and Pittet, 2002).

## Results

### Antibacterial Activity of Different *Xenorhabdus* and *Photorhabdus* Spp. Against *Enterococcus faecalis*

#### Overlay Bioassay

**Figure 1:**
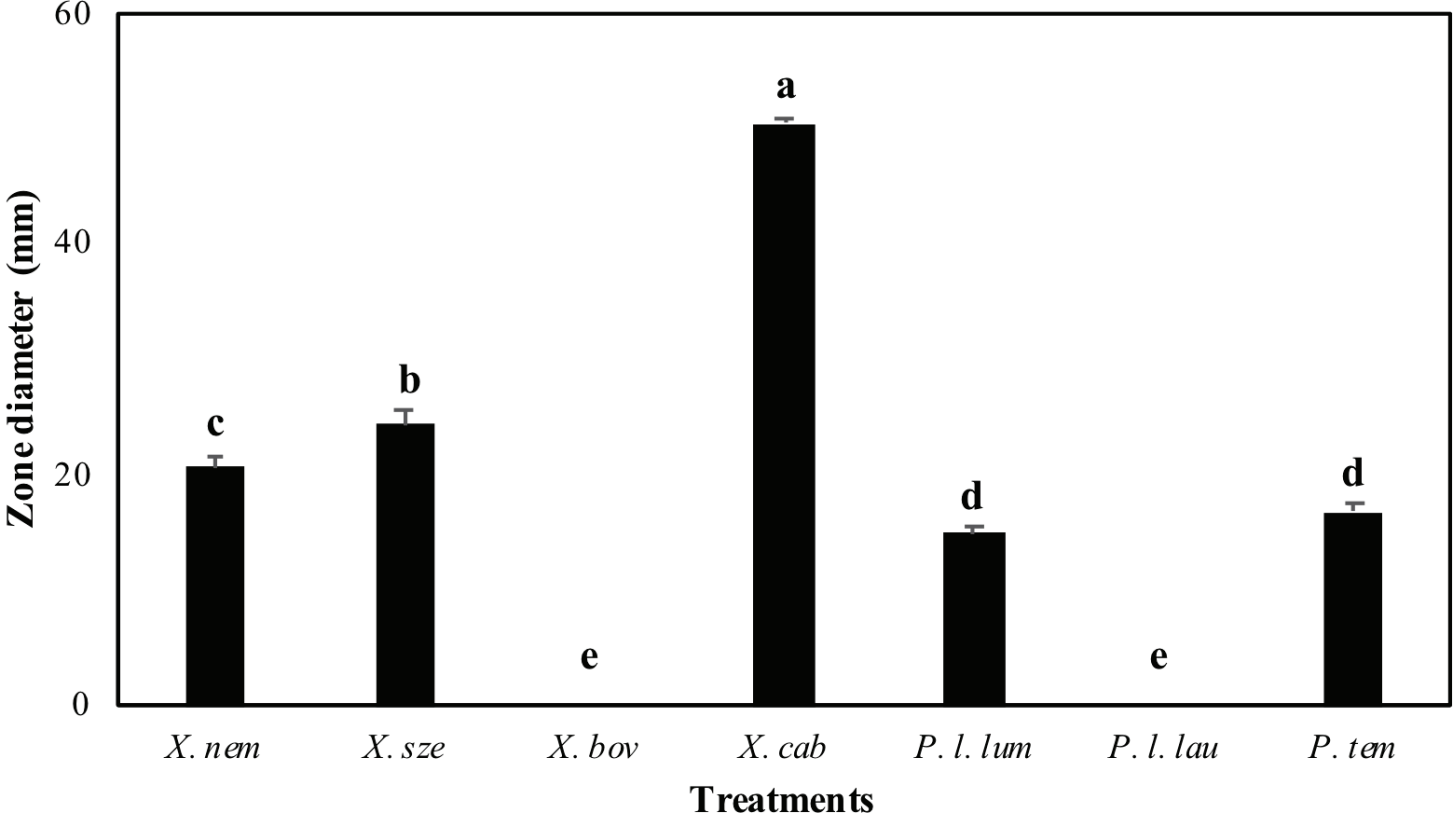
Inactivation zones (mm) resulting from overlay experiments using species of *Xenorhabdus* and *Photorhabdus* against *Enterococcus faecalis*. X. nem, *Xenorhabdus nematophilus*; X. sze, *X. szentirmaii*; X. bov, *X. bovienii*; X. cab, *X. cabanillasii*; P. l. lum, *Photorhabdus luminescens luminescens*; P. l. lau, *P. luminescens laumondii*; P. tem, *P. temperata*. Means indicated by the different lower-case letters on the bars are significantly different (*p* < 0.05).

The antibiotic production of *Xenorhabdus* and *Photorhabdus* against *E. faecalis* differed significantly among species (*F* = 527.73; df = 6, 202; *p* < 0.001) (Figure 1). *Xenorhabdus cabanillasii* had the most pronounced inactivation (50.4 mm) and *X. bovienii* and *P. luminescens laumondii* the lowest (Figure 1).

### Antibacterial Activity of Cell-Free *Xenorhabdus* or *Photorhabdus* Supernatants

All *Photorhabdus* and *X. bovienii* supernatants caused inactivation (based on lack of visible growth; MID) when undiluted supernatants were used (Supplementary Table S3). *Xenorhabdus nematophila* and *X. szentirmaii* caused inactivation at 20 and 40% proportions of supernatant including cultures, respectively. The greatest inactivation was achieved by *X. cabanillasii,* which showed inactivation even at a 1% concentration of supernatant. After the transfer of samples where no visually bacterial growth was observed on the blood-agar media, *E. faecalis* colonies were observed even at undiluted supernatants (100%) of *X. nematophila*, *X. bovienii,* and all *Photorhabdus* species (Supplementary Table S3). *Xenorhabdus szentirmaii* exhibited complete inactivation (MBD) only at 100% supernatant concentration. Similar to the visual inactivation results, we observed that the 5% supernatant of *X. cabanillasii* eliminated all cells of *E. faecalis* (Supplementary Table S3). *Enterococcus faecalis* proliferated in all positive controls, whereas no bacterial growth was observed in the negative control groups.

### Time-Dependent Inactivation of Cell-Free Supernatant

**Figure 2:**
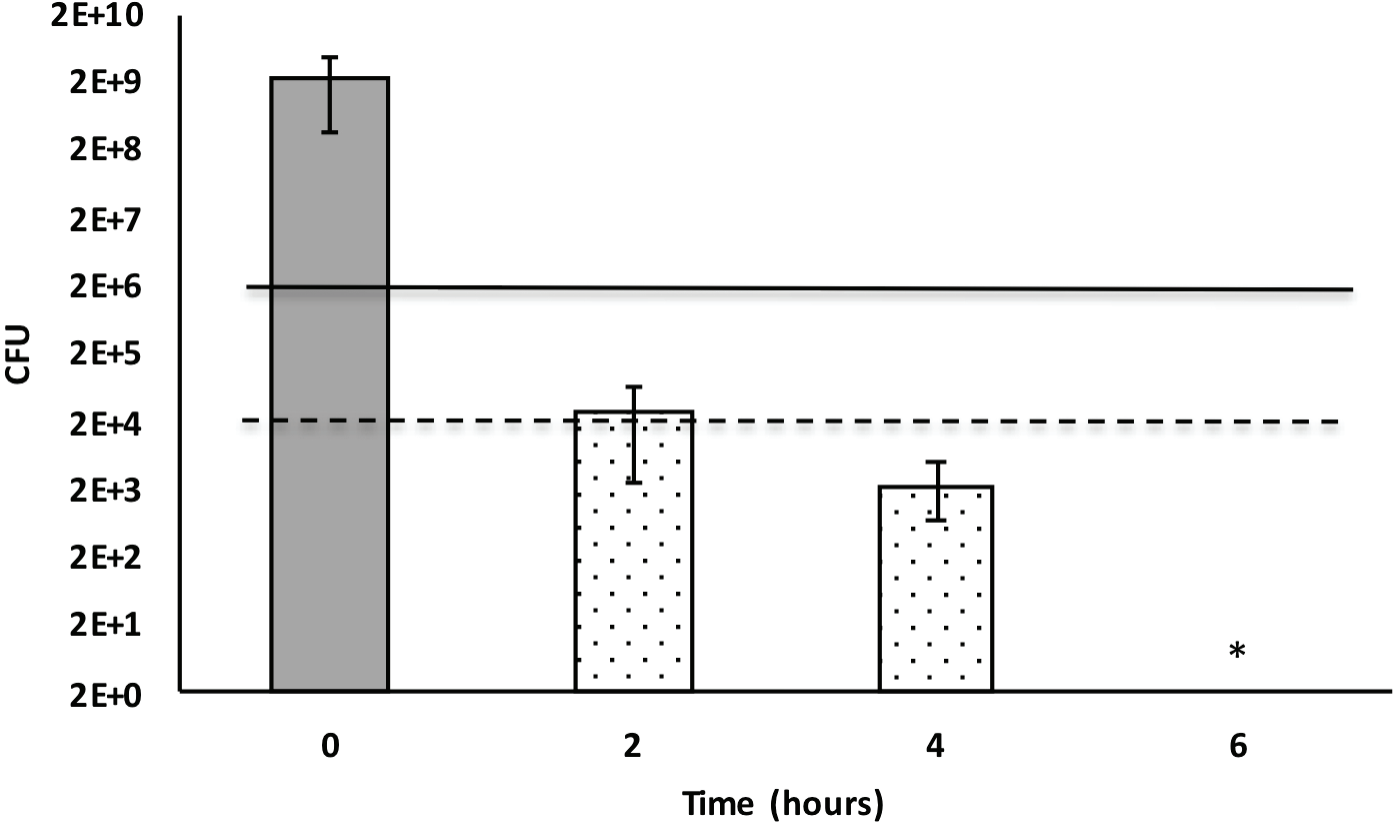
Time-dependent inactivation of *Enterococcus faecalis* cells incubated in 50% *Xenorhabdus cabanillasii* supernatant. Data in this figure are shown as CFU medians with 25 and 75% quartiles. Solid and dashed lines represent reductions of ≥3 and ≥ 5 log_10_ steps CFU, respectively. * indicates reduction below detection limit.

Bacterial culture media that included 50% *X. cabanillasii* supernatant showed inactivation starting from the time of the first subsample (2 h after inoculation) and the number of colonies of *E. faecalis* gradually decreased over time. The supernatant killed 99.999% of the bacteria after 2 h. Complete inactivation of *E. faecalis* occurred 6 h after the inoculation (Figure 2).

### Testing the Antibacterial Activity of *Xenorhabdus cabanillasii* Mutant Strains

**Figure 3:**
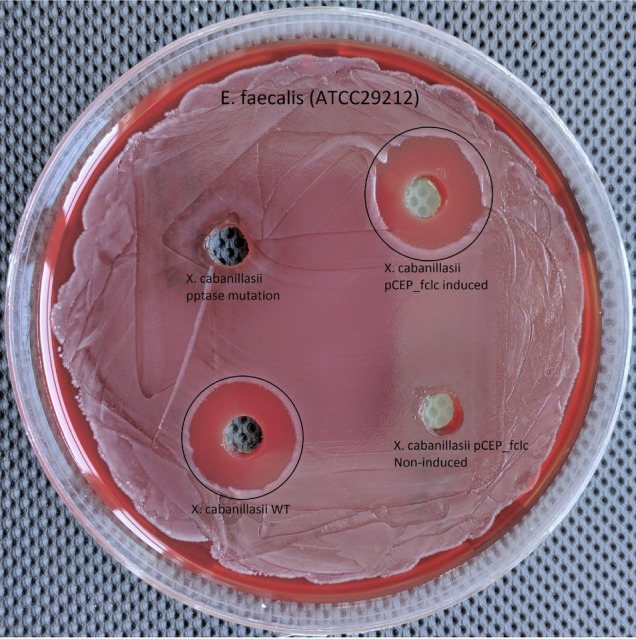
Comparison of wild-type, Δ*pptase*, pCEP*-fclC* induced, and pCEP*-fclC* non-induced mutant of *X. cabanillasii* against *Enterococcus faecalis.*

Wild-type and induced *fclC* promoter exchange mutant of *X. cabanillasii* supernatants exhibited average of 18 (±1.4) and 17.5 (±0.8) mm zone diameters, respectively. But, there was no inactivation circle around the wells of Δ*pptase* and non-induced *fclC* mutants (Figure 3).

### Identification of Bioactive Antibacterial Compound Produced by *Xenorhabdus cabanillasii*

**Figure 4:**
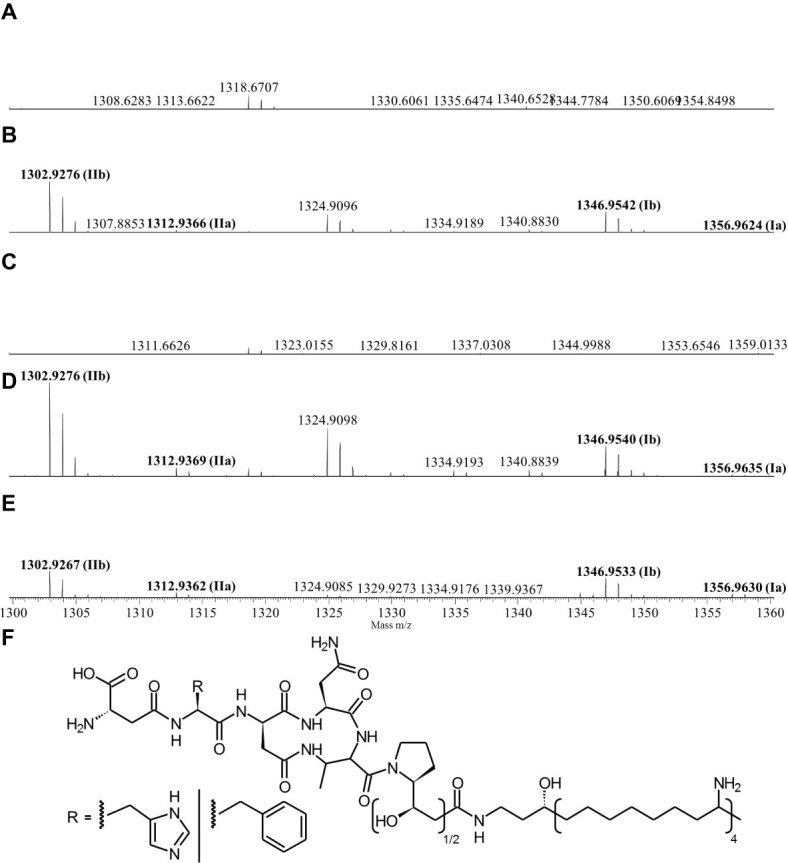
High-resolution MALDI-MS data of liquid cultures of *X. cabanillasii* ∆*ppt* **(A)**, *X. cabanillasii* JM26 WT **(B)**, *X. cabanillasii* P_Bad_-*fclC* (non-induced) **(C)**, *X. cabanillasii* P_Bad_-*fclC* (induced) **(D)**, *X. budapestensis* DSM 16342 **(E)**, and the corresponding fabclavine derivatives **(F)**. Highlighted in bold are signals that correspond with identified fabclavines in *X. budapestensis* (Fuchs et al., 2014). Cultures were grown for 72 at 30°C. MALDI-MS measurement was internally calibrated.

Since the Δ*pptase* mutant showed no more bioactivity (Figure 3), it can be postulated that the responsible compound is generated by a non-ribosomal peptide synthetase (NRPS) or a polyketide synthase (PKS). *In silico* analysis of the *X. cabanillasii* genome revealed multiple potential NRPS- and PKS-BGCs. Due to the known broad-spectrum bioactivity of zeamine/fabclavine, we focused on the fcl-homologous BGC (Fuchs et al., 2014; Masschelein et al., 2015a,b). We performed a promoter exchange in front of the first essential biosynthesis gene *fclC* and observed that the bioactive antibacterial compound is produced by this BGC (Figure 3) (Wenski et al., 2019). High-resolution MALDI-MS comparison revealed that the active compounds are the fabclavines Ia, Ib, IIa, and IIb, which were previously described for *X. budapestensis* DSM 16342 (Figure 4) as confirmed by the fragmentation pattern of signal 1302.92 (IIb) (Supplementary Table S4 and Supplementary Figure S1).

### Medicament Potential of the Antibacterial Compound in Dental Root Canals

**Figure 5:**
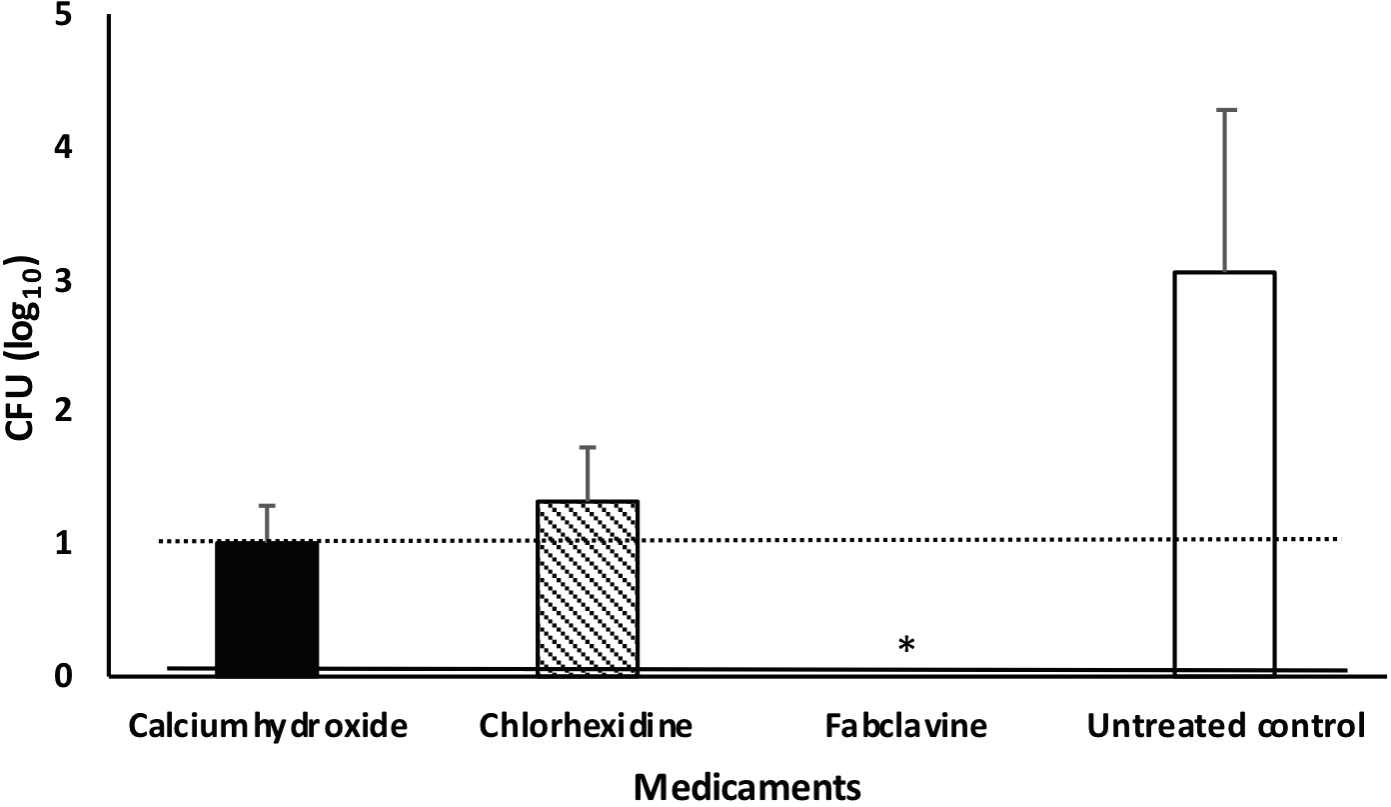
Median number of *Enterococcus faecalis* colony after treating dental root canals with different medicaments. Data were shown as CFU medians. Dotted and solid lines represent reductions of ≥2 and ≥ 3 log_10_ steps CFU, respectively. * indicates reduction below detection limit.

Fabclavine-rich supernatant exhibited the highest inactivation efficacy of ≥3 log_10_ steps CFU reduction, followed by calcium hydroxide paste (≤2 log_10_ step) (Figure 5).

If we considered no tolerance to any bacterial growth in root canals (even one cell), the fabclavine-rich supernatant completely eradicated *E. faecalis* in 63.6% of the treated teeth. The mean percentage of *E. faecalis*-free dental root canals after treatment was 45.5% for Ca(OH)_2_ and 18.2% for CHX. The median number of colonies were 0, 10, and 20 for fabclavine-rich supernatant, Ca(OH)_2_, and CHX, respectively. However, number of colonies in the control group was 1,120 CFU (Figure 5).

## Discussion

Our data showed that there were significant variations among the seven bacterial species in the production of effective antibacterial compounds against multidrug-resistant *E. faecalis*. Overall, the supernatant of *X. cabanillasii* exhibited the greatest inhibitory and bactericidal effect followed by supernatants of *X. nematophila* and *X. szentirmaii.* On the other hand, supernatants of *X. bovienii* and *P. luminescens laumondii* did not display any antibacterial efficacy against *E. faecalis*. Prior studies have reported variations among *Xenorhabdus* or *Photorhabdus* species (or strains) in the production of antimicrobial metabolites, and the efficacy of metabolites differed depending on the target organisms (Furgani et al., 2008; Fodor et al., 2010; Hazir et al., 2016). Furgani et al. (2008) reported that *X. cabanillasii* and *X. szentirmaii* produced larger diameter inhibitory zones than *X. nematophila* and *X. bovienii* against the primary mastitis pathogens *Staphylococcus aureus*, *Klebsiella pneumoniae*, and *Escherichia coli* when a cell-to-cell competition bioassay was conducted. In another study, *X. szentirmaii* produced a larger inhibitory zone (73.7 mm) than *X. cabanillasii* (60.7 mm) against Gram positive *S. aureus* (Fodor et al., 2010). However, in our overlay bioassay, cell-free supernatant of *X. cabanillasii* resulted in an inactivation zone of 50.4 mm, whereas *X. szentirmaii* exhibited an inactivation zone of 24.4 mm with *E. faecalis*. This discrepancy probably can be attributed to differences in the target organisms, or possibly strain differences in *X. szentirmaii* and *X. cabanillasii* used in the two different studies.

Previous studies demonstrated that *Xenorhabdus* and *Photorhabdus* secrete antimicrobial compounds (Furgani et al., 2008; Fodor et al., 2010; Hazir et al., 2016). Furgani et al. (2008) and Gualtieri et al. (2012) observed antibiotic activity when used not only as the purified antimicrobial compound, but also the cell-free supernatants of *Xenorhabdus* spp. Similarly, we observed antibacterial activity and determined MID and MBD values of *Xenorhabdus* and *Photorhabdus* in cell-free supernatants. The best MID and MBD values against *E. faecalis* were obtained from *X. cabanillasii* supernatant. Concentrations of 1 and 5% of *X. cabanillasii* supernatant were sufficient to inhibit bacterial growth (MID) and completely eliminate *E. faecalis* (MBD), respectively. Gualtieri et al. (2012) determined MBC values as the lowest concentration of antibiotic that resulted in 0.1% survival in the subculture. But we aimed for the complete inactivation of *E. faecalis* to prevent its re-colonization in the dental root canal. Therefore, our MBD values were designated according to full eradication. Our data clearly demonstrated that *X. cabanillasii* produce bactericidal molecule(s) rather than one with bacteriostatic effects, whereas other *Xenorhabdus* and *Photorhabdus* spp. tested in our study showed only a bacteriostatic effect.

To restrict the quantity of potential biosynthesis gene clusters responsible for the bactericidal compound, we compared the WT and Δ*pptase* mutant of *X. cabanillasii*. The loss of bioactivity of the Δ*pptase* mutant suggested the antibacterial compound against *E. faecalis* to be a natural product dependent on a PPTase as it is the case for typical NRPS- or PKS-derived natural products. Different biosynthetic gene clusters are known for *X. cabanillasii*, which could produce potential bioactive compounds like PAX-peptides, rhabdopeptides, or fabclavines (Tobias et al., 2017). The bioactivity of the mutant strain with a promoter exchange in front of the fabclavine-homologous gene cluster indeed confirmed that the bioactive compounds are fabclavines (Fuchs et al., 2014; Fodor et al., 2017). Biochemically, they are derived from a NRPS that produces a hexapeptide, which is elongated with one or two malonate units by a PKS and connected with an unusual polyamine (Fuchs et al., 2014; Wenski et al., 2019). Furthermore, our MALDI-MS and MS^2^ experiments confirmed that the fabclavines produced by *X. cabanillasii* are identical to the already described derivatives of *X. budapestensis* (Fuchs et al., 2014). It was reported that fabclavines Ia and Ib had a broad-spectrum bioactivity against different organisms such as *Micrococcus luteus, Escherichia coli, Bacillus subtilis*, *Saccharomyces cerevisiae, Trypanosoma cruzi, T. brucei,* and *Plasmodium falciparum* (Fuchs et al., 2014). Tobias et al. (2017) stated that except for *Photorhabdus asymbiotica* that might produce a shortened fabclavine derivative, other *Photorhabdus* species do not produce fabclavines, which were more widespread in *Xenorhabdus* strains. These data can explain why none of our tested *Photorhabdus* showed antibacterial efficacy against *E. faecalis.*

The success of endodontic treatment depends mainly on the complete inactivation of the infecting microorganisms from the root canal and prevention of reinfection. However, it is known that conventional root canal irrigants have limited action inside dentinal tubules beyond which viable bacteria are present (Gu et al., 2009). Our control data also showed that the irrigation of root canal with conventional irrigants (EDTA and NaOCl) was not enough to eradicate *E. faecalis* from dentinal tubules. Therefore, the use of intracanal medicaments between appointments is suggested for complete inactivation of bacteria before filling root canals (Bystrom et al., 1985; Pektas et al., 2013). Over the years, a number of synthetic antimicrobial agents have been employed as endodontic irrigants and medicaments against *E. faecalis*. Because of toxic and harmful side effects of common antibacterial agents and the increased antibiotic resistance to antimicrobial agents, a search for alternative agents that are non-toxic, affordable, and effective is needed.

Calcium hydroxide and CHX gel are known as the most effective intracanal medicaments and commonly used in dental practices (Haapasalo and Orstavik, 1987; Evans et al., 2003; Lakhani et al., 2017). However, they are not sufficient for the complete inactivation of *E. faecalis* from root canals in all cases (Siqueira, 2001; Evans et al., 2003; Mozayeni et al., 2014). The results of our study showed that 9 and 5 of 11 teeth treated with CHX and Ca(OH)_2_ were still contaminated with *E. faecalis*, respectively. Fabclavine-rich supernatant of *X. cabanillasii* exhibited more antimicrobial efficacy than CHX and Ca(OH)_2_ in root canals with complete inactivation from 7 of 11 teeth. Furthermore, Ca(OH)_2_ and CHX are formulated as paste and gluconate gel, respectively, to provide longer and better contact with microorganisms in root canals, whereas bacterial supernatant was in liquid form. This could be a possible reason why we did not obtain complete eradication in the root canals of all teeth (even though *X. cabanillasii* supernatant exhibited very strong bactericidal activity). We observed that some of the supernatant filled in root canal run off from the apex. Thus, it might be that the antibacterial compound does not reach *E. faecalis* hidden deep in the dentinal tubules. Accordingly, it will be useful to test the efficacy of formulated fabclavine-rich supernatant in future studies.

Fabclavines show very strong antimicrobial effects against both prokaryotic and eukaryotic pathogens and therefore they might also display adverse effects to host cells (Fuchs et al., 2014; Fodor et al., 2017; Tobias et al., 2017).

Our data clearly revealed that the fabclavine in the supernatant of *X. cabanillasii* has strong antibacterial activity against *E. faecalis*. Here we used fabclavine-rich supernatant as intracanal medicament for simplicity and it was highly effective against multidrug-resistant *E. faecalis.* Fabclavines might not display equivalent efficiencies against all bacterial species especially in primary endodontic infections which are polymicrobial. In this type of situation, they can be combined with other antibiotics which would lead to synergistic effects on pathogens while simultaneously reducing the potential adverse side effects (Fuchs et al., 2014).

We also compared the efficacy of cell-free supernatant of *X. cabanillasii* against multidrug-resistant V583 (ATCC 700802), antibiotic susceptible (ATCC 29212) and a clinic isolate (obtained from root canal of a patient) of *E. faecalis* using overlay bioassay method and there was no difference among the zone diameters (data were not shown in the manuscript). This indicates that the antibacterial mechanism of fabclavine derivatives has a different mode of action than commonly used traditional antibiotics. Based on the structural similarities to the (pre-)zeamines, we assume a similar mode of action (Masschelein et al., 2015a). Zeamines also show a broad-spectrum bioactivity against a wide variety of organisms, which is probably caused by a membrane disruptive mode of action (Masschelein et al., 2015b).

In conclusion, the data of the induced and non-induced fabclavine promoter exchange mutants clearly show that fabclavine derivatives are bioactive compounds responsible for the bactericidal effect. Although commonly used synthetic intracanal medicaments CHX gel and Ca(OH)_2_ paste do not eradicate infected root canals in all cases, they are commonly used as medicaments in root canals. Instead, purified and formulated fabclavine derivatives have a great potential to be used as intracanal medicament against dental root canal infections. Further studies with fabclavine-rich derivatives at different concentrations, different formulations and combine applications with other medicaments are needed to test against potential root canal pathogens in *in vitro* and *in vivo* bioassays. Of course one must determine the toxicity of fabclavines against eukaryotic cells at the applied concentration, since they also show some bioactivity against cell lines (Fuchs et al., 2014). However, the large number of fabclavine derivatives found in other *Xenorhabdus* strains might enable the identification of more specific derivatives with less toxicity.

Though *Enterococcus faecalis* is a commensal organism of humans, it is the third most common pathogen isolated from human bloodstream infections (Karchmer, 2000). It can also cause endocarditis; meningitis; and nosocomial, urinary tract, and wound infections (Kau et al., 2005). The efficacy of fabclavine derivatives obtained from *X. cabanillasii* can also be evaluated against these infections in the future.

## Data Availability Statement

All datasets generated for this study are included in the article/Supplementary Material.

## Ethics Statement

The studies involving human participants were reviewed and approved by Adnan Menderes University, Local Ethical Committee. The patients/participants provided their written informed consent to participate in this study.

## Author Contributions

SH and HD designed the research. HD, HC, DU, and MT carried out the research, collected the data, and contributed to data analyses. HB and SW generated promoter exchanged mutant strains. NA and SY assisted with the experiments. SH and HB wrote the paper. All authors discussed the results and commented on the manuscript.

### Conflict of Interest

The authors declare that the research was conducted in the absence of any commercial or financial relationships that could be construed as a potential conflict of interest.
