# Supplementary material for: Nematode-Associated Bacteria: Production of Antimicrobial Agent as a Presumptive Nominee for Curing Endodontic Infections Caused by Enterococcus faecalis
Source: Front Microbiol. 2019 Nov 22;10:2672. doi: 10.3389/fmicb.2019.02672 (PMC6882856; doi:10.3389/fmicb.2019.02672)
Supplement: Supplementary file 1 [file Table_1.DOCX]

***Supplementary Material***

**Supplementary Table S1**. The bacterial species and subspecies and their nematode host species (and strain where known) used in the assay against *Enterococcus faecalis*.

| **Mutualistic bacteria** | **Entomopathogenic nematode** | **Nematode strain** |
| --- | --- | --- |
| *Xenorhabdus nematophila* | *Steinernema carpocapsae* | Rize |
| *Xenorhabdus szentirmaii* | *Steinernema rarum* | 17C&E |
| *Xenorhabdus bovienii* | *Steinernema feltiae* | 09-38 |
| *Xenorhabdus cabanillasii* | *Steinernema riobrave* | 355 |
| *Photorhabdus luminescens luminescens* | *Heterorhabditis bacteriophora* | 09-20 |
| *Photorhabdus luminescens laumondii* | *Heterorhabditis bacteriophora* | 48-02 |
| *Photorhabdus temperata* | *Heterorhabditis megidis* | UCDavis |

**Supplementary Table S2.** Used strains and plasmids.

| Strain/ plasmid | Description | Origin |
| --- | --- | --- |
| *X. cabanillasii* JM26 | Wild type strain | [3] |
| *X. budapestensis*  DSM 16342 | Wild type strain | [4] |
| *X. cabanillasii* ∆*ppt* | Deletion of phosphopantetheinyl transferase (Xcab_04003) | This work |
| *X. cabanillasii* PBad-*fclC* | Promotorexchange in front of fabclavine-homologeous cluster | This work |
| *E. coli* S17-1λpir | Conjugation strain | [5] |
| *E. coli* ST18 | Conjugation strain | [6] |
| pDS132 | Deletion vector | [7] |
| pCEP_km | Promotorexchange vector | [2] |
| pSW_∆*ppt* | pDS132 with up- and downstream regions of Xcab_04003 | This work |
| pSW_P_Bad_-*fclC* | pCEP_km with about first 1000 bp of Xcab_02060 | This work |

**Supplementary Table S3**. Antibiotic activity (maximum inhibiting dilution; MID and maximum bactericidal dilution; MBD) of 6-day-old cell-free cultures of *Xenorhabdus* and *Photorhabdus* spp. against *Enterococcus faecalis*.

| **Bacterial Supernatant** | **Maximum inhibiting dilution (MID) of bacterial supernatant (%)** | **Maximum bactericidal dilution (MBD) of bacterial supernatant (%)** |
| --- | --- | --- |
| *Xenorhabdus nematophila* | 20 | - |
| *Xenorhabdus szentirmaii* | 40 | 100 |
| *Xenorhabdus bovienii* | 100 | - |
| *Xenorhabdus cabanillasii* | 1 | 5 |
| *Photorhabdus luminescens luminescens* | 100 | - |
| *Photorhabdus luminescens laumondii* | 100 | - |
| *Photorhabdus temperata* | 100 | - |

(-) indicating no complete inhibition of *E. faecalis* at any supernatant doses.

**Supplementary Table S4.** High resolution MALDI-MS masses for the fabclavine derivatives detected in liquid cell cultures of *X. budapestensis* DSM 16342 (Figure 4, **E**) and *X. cabanillasii* P_Bad_-*fclC* (ind.) (Figure 4, **D**). Cultures were grown for 72 h h at 30°C. MALDI-MS measurement was internally calibrated.

|  |  | *X. budapestensis* | | *X. cabanillasii* P_Bad_-*fclC* (ind.) | |
| --- | --- | --- | --- | --- | --- |
| Fabclavine | calc m/z | det m/z | error [∆ppm] | det m/z | error [∆ppm] |
| Ia | 1356.9588 | 1356.9630 | +2.773 | 1356.9635 | +3.141 |
| Ib | 1346.9493 | 1346.9533 | +2.610 | 1346.9540 | +3.166 |
| IIa | 1312.9324 | 1312.9362 | +2.435 | 1312.9369 | +2.923 |
| IIb | 1302.9230 | 1302.9267 | +2.425 | 1302.9276 | +3.116 |


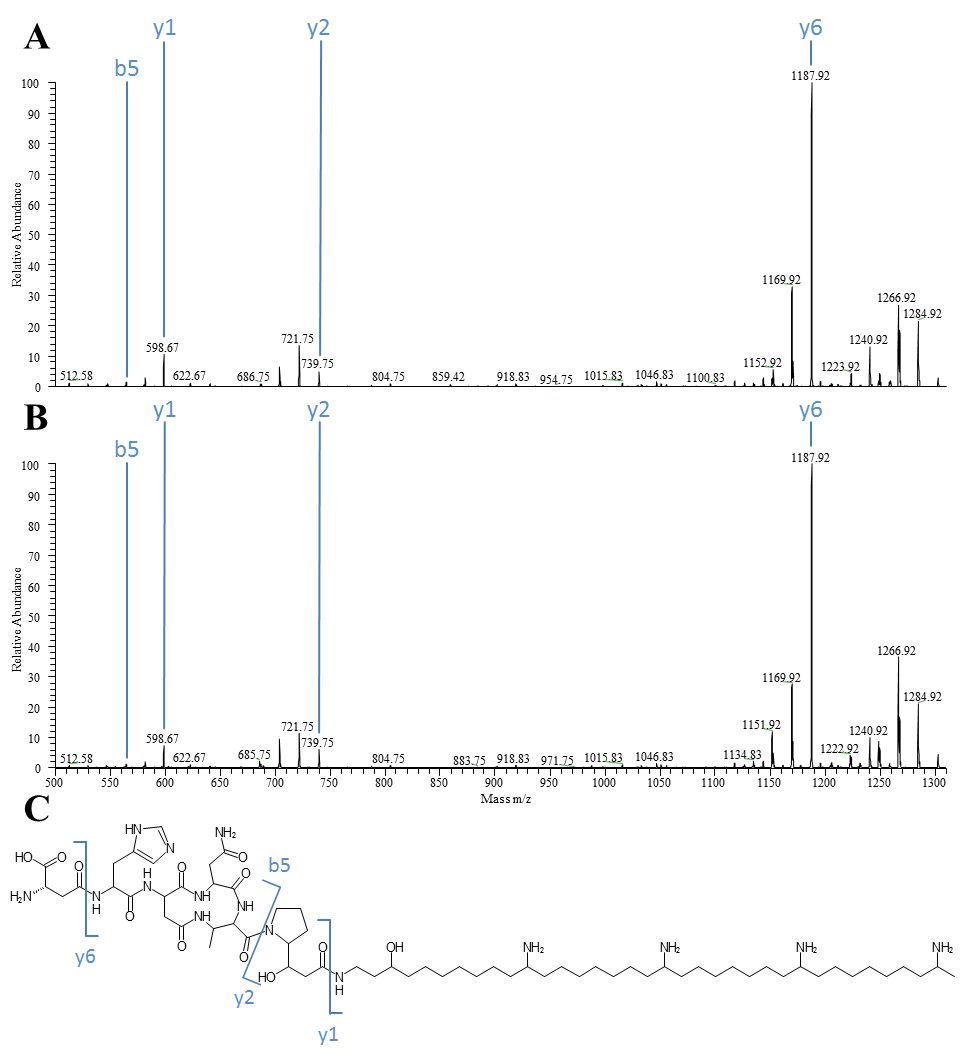


**Supplementary Figure S1.** MALDI-MS^2^-spectras of signal 1302,92 (IIb) in liquid cell cultures of *X. cabanillasii* P_Bad_-*fclC* (ind.) (**A**), *X. budapestensis* DSM 16342 (**B**) and structure of fabclavine IIb with marked fragment-ions (**C**) [1]. Marked in blue are signals which corresponds with identified fabclavine fragment-ions in *X. budapestensis* [1]. Cultures were grown for 72 h h at 30°C.

**References of supporting information:**

[1] Fuchs, S. W., Grundmann, F., Kurz, M., Kaiser, M., Bode, H. B. (2014). Fabclavines: bioactive peptide-polyketide-polyamino hybrids from *Xenorhabdus*. *Chembiochem*. 15(4), 512-516.

[2] Bode, E., Brachmann, A. O., Kegler, C., Simsek, R., Dauth, C., Zhou, Q., et al. (2015) Simple "on-demand" production of bioactive natural products. *Chembiochem*. 16(7), 1115-1119.

[3] Tailliez, P., Pagès, S., Ginibre, N., Boemare, N. (2006). New insight into diversity in the genus *Xenorhabdus*, including the description of ten novel species. *Int. J. Syst. Evol. Microbiol*. 56, 2805-2818.

[4] Lengyel, K., Lang, E., Fodor, A., Szállás, E., Schumann, P., Stackebrandt, E. (2005). Description of four novel species of *Xenorhabdus*, family Enterobacteriaceae: *Xenorhabdus budapestensis* sp. nov., *Xenorhabdus ehlersii* sp. nov., *Xenorhabdus innexi* sp. nov., and *Xenorhabdus szentirmaii* sp. nov. *Syst. Appl. Microbiol*. 28(2), 115-122.

[5] Simon, R., Priefer, U., Pühler, A. (1983). A Broad Host Range Mobilization System for *In Vivo* Genetic Engineering: Transposon Mutagenesis in Gram Negative Bacteria. Bio/Technology volume 1, pages 784–791.

[6] Thoma, S., and Schobert, M. (2009). An improved Escherichia coli donor strain for diparental mating. *FEMS Microbiol. Lett.* 294(2), 127-132.

[7] Philippe, N., Alcaraz, J. P., Coursange, E., Geiselmann, J., Schneider, D. (2004). Improvement of pCVD442, a suicide plasmid for gene allele exchange in bacteria. *Plasmid.* 51(3), 246-255.
